# The trend of lymphoma incidence in China from 2005 to 2017 and lymphoma incidence trend prediction from 2018 to 2035: a log-linear regression and Bayesian age-period-cohort analysis

**DOI:** 10.3389/fonc.2024.1297405

**Published:** 2024-05-29

**Authors:** Kangqian Lin, Jianjiang Shao, Yuting Cao, Lijun Lu, Peng Lei, Xiaohong Chen, Mengwei Tong, Yaping Lu, Yizhong Yan, Lei Zhang, Xin Pan, Weixia Nong

**Affiliations:** ^1^ Department of Preventive Medicine, School of Medicine, Shihezi University, Shihezi, Xinjiang, China; ^2^ Department of Hematology, The First Affiliated Hospital of Shihezi University, Shihezi, Xinjiang, China; ^3^ Clinical Laboratory, The First Affiliated Hospital of Shihezi University, Shihezi, Xinjiang, China; ^4^ National Hematology Clinical Research Center Xinjiang Production and Construction Corps Branch Center, Shihezi, Xinjiang, China

**Keywords:** lymphoma, incidence, joinpoint regression model, age-period-cohort model, Bayesian age-period-cohort model

## Abstract

**Objectives:**

The aims of this study were to explore the incidence characteristics and trend prediction of lymphoma from 2005 to 2035, and to provide data basis for the prevention and control of lymphoma in China.

**Method:**

The data on lymphoma incidence in China from 2005 to 2017 were obtained from the Chinese Cancer Registry Annual Report. The Joinpoint regression model was used to calculate annual percentage change (APC) and average annual percentage change (AAPC) to reflect time trends. Age–period–cohort models were conducted to estimate age, period, and cohort effects on the lymphoma incidence. A Bayesian age–period–cohort model was used to predict lymphoma incidence trends from 2018 to 2035.

**Results:**

From 2005 to 2017, the incidence of lymphoma was 6.26/100,000, and the age-standardized incidence rate (ASIR) was 4.11/100,000, with an AAPC of 1.4% [95% confidence interval (CI): 0.3%, 2.5%]. The ASIR was higher in men and urban areas than in women and rural areas, respectively. The age effect showed that the incidence risk of lymphoma increased with age. In the period effect, the incidence risk of lymphoma in rural areas decreased first and then increased with 2010 as the cutoff point. The overall risk of lymphoma incidence was higher in the cohort before the 1970–1974 birth cohort than in the cohort after. From 2018 to 2035, the lymphoma incidence in men, women, and urban areas will show an upward trend.

**Conclusion:**

From 2005 to 2017, the incidence of lymphoma showed an increasing trend, and was different in regions, genders, and age groups in China. It will show an upward trend from 2018 to 2035. These results are helpful for the formulation and adjustment of lymphoma prevention, control, and management strategies, and have important reference significance for the treatment of lymphoma in China.

## Introduction

Since the 20th century, the disease spectrum has changed in most countries around the world. The source of disease burden has shifted from infectious diseases to chronic non-communicable diseases (NCDs), such as cardiovascular diseases and cancers, which have become the primary diseases affecting human health and life. According to the World Health Organization (WHO), NCDs kill an estimated 41 million people every year, equivalent to 74% of all deaths worldwide (1). Among NCDs, cancer ranks second in the number of deaths after cardiovascular diseases, accounting for 22.7% of all NCD deaths (1). According to the latest report of WHO’s International Agency for Research on Cancer, by 2020, there will be an estimated 19.3 million new cancer cases and nearly 10 million cancer deaths worldwide (2). Cancer has become one of the main causes of human incidence and death worldwide (3, 4).

Among the many types, lymphoma is one of the relatively common cancers. Lymphoma is a cancer that originates in hematopoietic and lymphoid tissues. According to the Global Cancer Report 2020, there were 627,400 (3.2%) new cases of lymphoma worldwide in 2020, an increase of 85.98% compared with 2000; the number of global lymphoma deaths in 2020 was 283,200 (2.8%), an increase of 47.8% from 2000 (2, 5). In 2020, there were approximately 101,400 new cases of lymphoma in China, accounting for 16.16% of the global lymphoma incidence; approximately 47,000 people died of lymphoma in China, accounting for 16.60% of the global lymphoma deaths (6). Lymphoma is still a health hazard and a public health problem that cannot be ignored.

Analyzing trends and predictions of lymphoma incidence could provide key clues to guide future epidemiological studies. This study collected national, gender, and region-specific lymphoma incidence data from the Chinese Cancer Registry Annual Report. The Joinpoint regression model was used to reflect the trend in lymphoma incidence from 2005 to 2017, and the age–period–cohort model was used to analyze the impact of age, period, and cohort effects on the risk of lymphoma. Using the Bayesian age–period–cohort (BAPC) model to predict the incidence trend of lymphoma from 2018 to 2035 could provide a reference for the adjustment of the prevention and control policy of lymphoma in the future, important basic data for the control of lymphoma in China, and a scientific basis for the treatment of lymphoma.

## Materials and methods

### Data sources

The data on the incidence and age-standardized incidence rate (ASIR) of lymphoma in China from 2005 to 2017 (7) came from the “Chinese Cancer Registry Annual Report, 2008–2020” (8–20). Lymphoma cases were identified according to the International Classification of Diseases (ICD-10/C81–85, C88, C90, C96); non-Hodgkin’s lymphoma, Hodgkin’s lymphoma, malignant immunoproliferative diseases, and malignant plasma cell neoplasms are included (21). The raw data were collected from the cancer registries of 31 provinces (autonomous regions and municipalities) and Xinjiang Production and Construction Corps, which is the only source of nationwide cancer data, and its coverage is still evolving and expanding, and is currently based on a sample of 10%–35% of the national population. In addition, the data are reviewed, evaluated, and organized for analysis in accordance with the Guidelines for Tumor Registration in China and the requirements of the International Association of Cancer Registries/International Agency for Research on Cancer (IARC). The registry procedure and things like coding practice are consistent across different areas and throughout the study period. The data quality evaluation used the indicators of the percentage of morphological verification of diagnosis, percentage of death certificate only, and the mortality/incidence ratios to evaluate the quality of data reporting (22). The average proportion of the indicators from 2005 to 2017 were 67.83, 2.11, and 0.62, respectively. The data quality of this database is high, and all the indicators are in line with the evaluation standard, which can truly reflect the cancer prevalence, and the data are reliable.

### Statistical analysis

#### Joinpoint regression model

The Joinpoint regression model was used to calculate the annual percentage change (APC), average annual percentage change (AAPC), and their 95% confidence interval (CI) to conduct trend analysis on the incidence of lymphoma. The detailed introduction has been described in our group’s previous study (23).

#### Age–period–cohort model

The age–period–cohort model was conducted to analyze the age, period, and cohort effects on lymphoma incidence. The detailed introduction has been described in our group’s previous study (23).

#### Bayesian age–period–cohort model

The BAPC model is based on the age–period–cohort model, which describes trends in disease by considering the effects of age, period, and cohort on the incidence. Using the age–period–cohort model, future incidence can be predicted. However, owing to the linear relationship between age, period, and cohort factors in the model, parameter estimation is difficult, so a Bayesian model is added. According to the Bayesian formula, the prior information of unknown parameters can be combined with the sample information to obtain the posterior information, and then the unknown parameters can be inferred according to the posterior information. The commonly used Integrated Nested Laplace Approximation (INLA) algorithm for model estimation directly approximates the posterior marginal distribution, avoiding mixing and convergence problems (24, 25). INLA operates as a precise framework within the Bayesian statistical approach, utilizing a blend of analytical methods and numerical integration techniques to obtain highly precise deterministic estimates for the posterior distributions of interest. A significant advantage of employing INLA is its rapid computational efficiency, which is particularly beneficial when dealing with expansive and intricate models. Additionally, INLA, as a deterministic procedure, is not prone to the issues of sluggish convergence and inadequate mixing that can plague other algorithms (26). The expression is:


$$f(\alpha |k_{\alpha })\propto k_{\alpha }^{\frac{I-2}{2}}\exp(-\frac{k_{\alpha }}{2}\sum {\;}_{i=3}^{I}{\left(\alpha_{i}-2\alpha_{i-1}+\alpha_{i-2}\right)}^{2})=k_{\alpha }^{\frac{I-2}{2}}\exp(-\frac{1}{2}\alpha^{T}Q\alpha )$$

where *k* is the precision parameter; *Q* is the second difference penalty term; and *α_i_* (*i*=1, …, *I*) is the age effect.

Population forecasts were derived from the World Population Prospects 2022 (27), the 27th version of the official population estimates and forecasts developed by the United Nations. Among them, in order to evaluate the performance of the BAPC model in predicting the incidence rates of lymphoma in China (i.e., according to gender and region) and the accuracy of the prediction, a retrospective prediction of the 2005–2017 data was performed by the BAPC model. The actual observations and the prediction result values were compared to each other with the three indexes of the mean absolute error, mean absolute percentage error, and the fitting accuracy (equal to 1 − mean absolute percentage error), which were used to evaluate the effectiveness of the forecasts; a fitting accuracy of 80% or more was considered good (28, 29).

Joinpoint regression analysis was performed using the Joinpoint Regression Program 5.0.2 software. In addition, with the Joinpoint Regression Program 5.0.2 software update, the model utilizes the Weighted Bayesian Information Criterion for the number, location, and *p*-value of the turning points. With the increased processing speed provided by the Weighted Bayesian Information Criterion approach, Joinpoint now allows a default setting of six or seven maximum number of Joinpoints, depending on the number of data points. The age–period–cohort model was conducted using the online web page analysis tool (30). BAPC and INLA packages of R 4.3.1 software were used to create a BAPC model. *p* < 0.05 was regarded as a statistically significant difference.

## Results

### The incidence and changing trends of lymphoma in China from 2005 to 2017

From 2005 to 2017, a total of 153,280 new cases of lymphoma were reported in Chinese cancer registration areas, including 88,902 men (58.0%) and 64,378 women (42.0%), with 98,807 cases (64.5%) in urban areas and 54,473 cases (35.5%) in rural areas. The incidence rate was 6.26/100,000 (men 7.17/100,000, women 5.33/100,000; urban 7.46/100,000, rural 4.85/100,000), and the ASIR was 4.11/100,000 (men 4.84/100,000, women 3.40/100,000; urban 4.69/100,000, rural 3.08/100,000). The incidence and ASIR were higher in men and urban areas (**Table 1**).

**Table 1:** Incidence of lymphoma in Chinese cancer registration areas from 2005 to 2017 (/100,000).

Indexes	National			Men			Women			Urban			Rural		
	Cases	Rates	ASIR	Cases	Rates	ASIR	Cases	Rates	ASIR	Cases	Rates	ASIR	Cases	Rates	ASIR
Incidence															
2005	3,381	6.16	3.71	1,990	7.15	4.47	1,391	5.13	2.98	2,880	7.08	4.16	501	3.52	2.39
2006	3,829	6.43	3.93	2,262	7.54	4.71	1,567	5.30	3.21	3,328	7.15	4.29	501	3.85	2.68
2007	3,625	6.06	3.57	2,102	6.95	4.17	1,523	5.15	3.01	3,026	6.78	3.91	599	3.94	2.64
2008	4,771	7.12	4.04	2,759	8.28	4.74	2,012	6.13	3.37	4,188	8.03	4.45	583	4.17	2.52
2009	5,713	6.68	3.75	3,332	7.71	4.46	2,381	5.64	3.05	4,718	8.21	4.47	995	3.56	2.18
2010	7,427	5.96	4.31	4,387	6.96	5.18	3,040	4.94	3.48	5,833	7.29	5.11	1,594	3.57	2.76
2011	8,888	6.10	4.39	5,187	7.04	5.21	3,701	5.13	3.60	6,389	7.30	5.01	2,499	4.29	3.35
2012	11,351	5.73	4.12	6,510	6.49	4.80	4,841	4.96	3.47	7,222	7.19	4.89	4,129	4.23	3.25
2013	13,984	6.17	4.32	8,133	7.08	5.11	5,851	5.24	3.55	8,559	7.67	5.05	5,425	4.72	3.53
2014	17,331	6.01	4.19	10,043	6.87	4.89	7,288	5.13	3.51	10,532	7.31	4.85	6,799	4.72	3.47
2015	20,655	6.44	4.41	12,042	7.40	5.18	8,613	5.45	3.66	11,719	7.60	4.98	8,936	5.36	3.84
2016	24,145	6.33	4.30	13,887	7.17	4.98	10,258	5.46	3.62	14,314	7.43	4.87	9,831	5.20	3.67
2017	28,180	6.46	4.34	16,268	7.36	5.07	11,912	5.54	3.63	16,099	7.55	4.91	12,081	5.42	3.78
Total	153,280	6.26	4.11	88,902	7.17	4.84	64,378	5.33	3.40	98,807	7.46	4.69	54,473	4.85	3.08

ASIR, age-standardized incidence rate.

**Figure 1:**
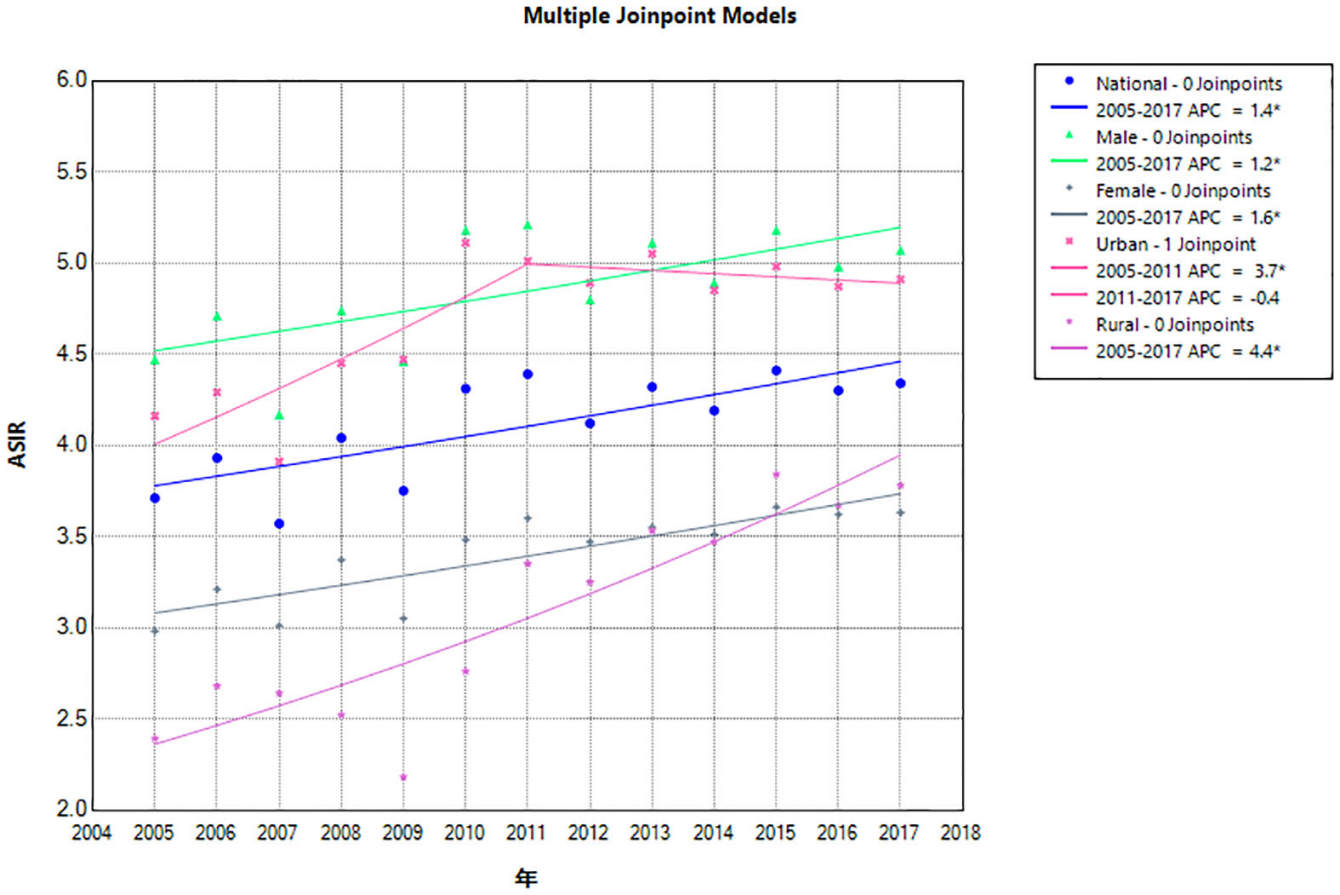
Joinpoint regression of lymphoma incidence in China from 2005 to 2017. APC, annual percentage change; ASIR, age-standardized incidence rate.

From 2005 to 2017, the ASIR of lymphoma in China increased from 3.71/100,000 in 2005 to 4.34/100,000 in 2017, AAPC = 1.4% (95% CI: 0.3%, 2.5%). For men, the ASIR of lymphoma increased from 4.47/100,000 in 2005 to 5.07/100,000 in 2017, AAPC = 1.2% (95% CI: 0.1%, 2.2%). For women, the ASIR increased from 2.98/100,000 in 2005 to 3.63/100,000 in 2017, AAPC = 1.6% (95% CI: 0.6%, 2.6%). By region, the ASIR of urban lymphoma increased from 4.16/100,000 in 2005 to 4.91/100,000 in 2017, AAPC = 1.7% (95% CI: 0.7%, 2.7%), of which, from 2005 to 2011, the APC was 3.7% (95% CI: 2.2%, 9.9%) and −0.4% (95% CI: −5.3%, 1.1%) in 2011–2017. For rural areas, it increased from 2.39/100,000 in 2005 to 3.78/100,000 in 2017, AAPC = 4.4% (95% CI: 2.8%, 6.3%). The growth rate of the ASIR of lymphoma in rural areas was 2.59 times that of urban areas, but it was lower than that in urban areas (**Tables 1**, **2**; **Figure 1**).

**Table 2:** Trends in lymphoma incidence in China from 2005 to 2017 (%).

Indexes	National	Men	Women	Urban	Rural
Periods	2005–2017	2005–2017	2005–2017	2005–2011	2005–2017
APC (95% CI)	1.4* (0.3, 2.5)	1.2* (0.1, 2.2)	1.6* (0.6, 2.6)	3.7* (2.2, 9.9)	4.4* (2.8, 6.3)
Periods				2011–2017	
APC (95% CI)				−0.4 (−5.3, 1.1)	
AAPC (95% CI)	1.4* (0.3, 2.5)	1.2* (0.1, 2.2)	1.6* (0.6, 2.6)	1.7* (0.7, 2.7)	4.4* (2.8, 6.3)

*Indicates that the APC is significantly different from zero at the alpha = 0.05 level. *Indicates that the AAPC is significantly different from zero at the alpha = 0.05 level. AAPC, average annual percentage change; APC, annual percent change; 95% CI, 95% confidence interval.

### Age–period–cohort model analysis

The age effect of lymphoma incidence risk was consistent across the country, different genders, and different regions. Among people aged 0–45 years, the risk of lymphoma slowly increased with age. In people aged 45–75 years, the risk of lymphoma increased with age. After the age of 75 years, it gradually decreased.

**Figure 2:**
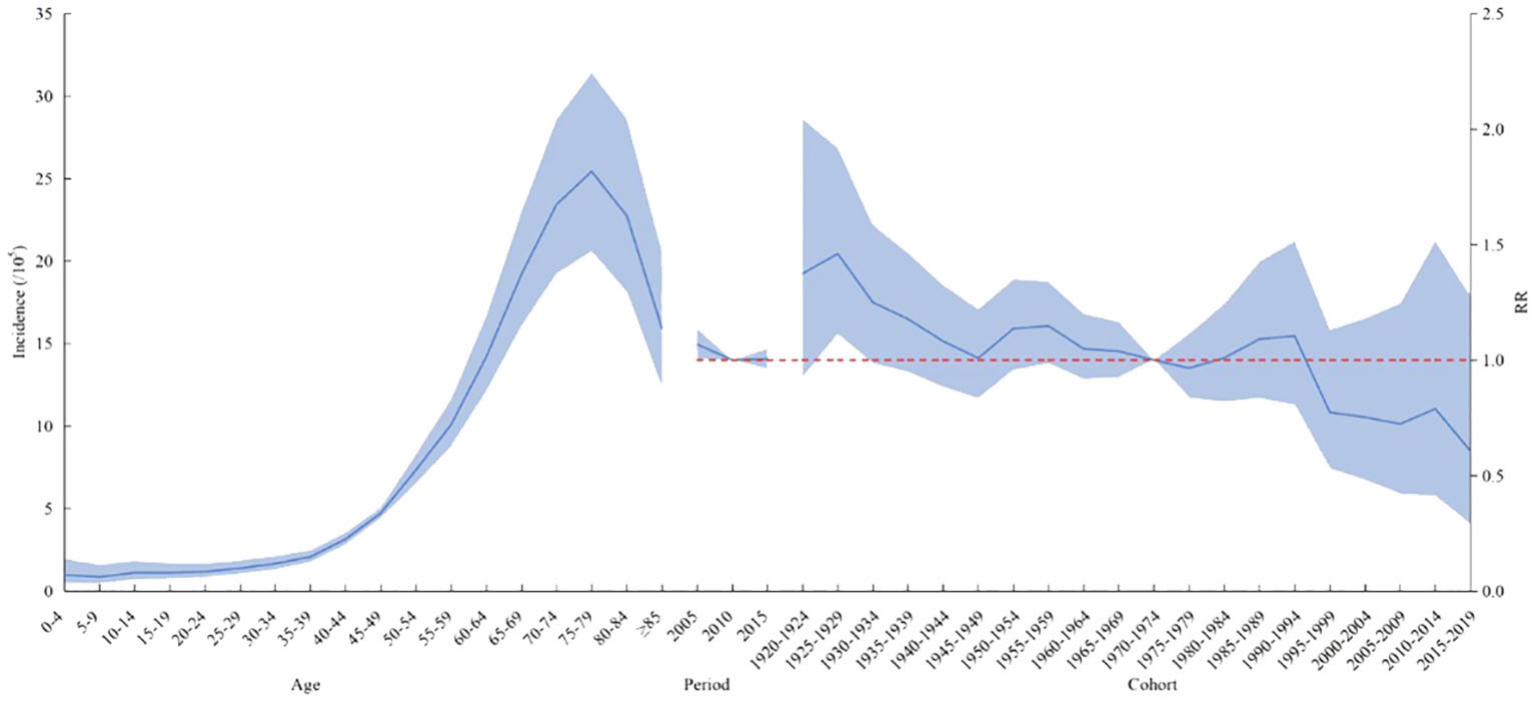
Age–period–cohort analysis of the incidence of lymphoma in China from 2005 to 2017. RR, risk ratio. The blue portion indicates the 95% confidence intervals, and the shaded bands represent the intervals in an increment or decrement of 10%.

**Figure 3:**
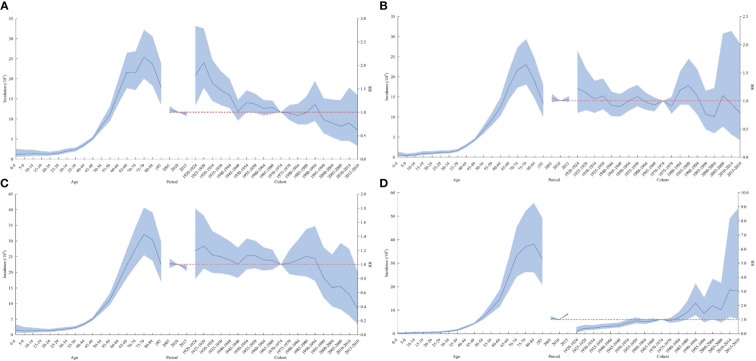
Age–cohort analysis of lymphoma incidence rates among men **(A)**, women **(B)**, urban **(C)**, and rural areas **(D)** in China from 2005 to 2017. RR, risk ratio. The blue portion indicates the 95% confidence intervals, and the shaded bands represent the intervals in an increment or decrement of 10%.

In terms of the period effect, the risk of lymphoma in the national level, in men, and in urban areas gradually decreased over time, while the lymphoma risk in women and rural areas decreased and then increased from 2010 as the cutoff point. The overall risk of lymphoma incidence was higher in the cohort before the 1970–1974 birth cohort than in the cohort after. The cohort effect trends for lymphoma risk in men, women, and urban areas were generally consistent nationally but reversed in rural areas. The cohort effect of the cohort group born after 1970–1974 was higher than that of the previous cohort group. The overall trend was increasing (**Figures 2**, **3**).

**Figure 4:**
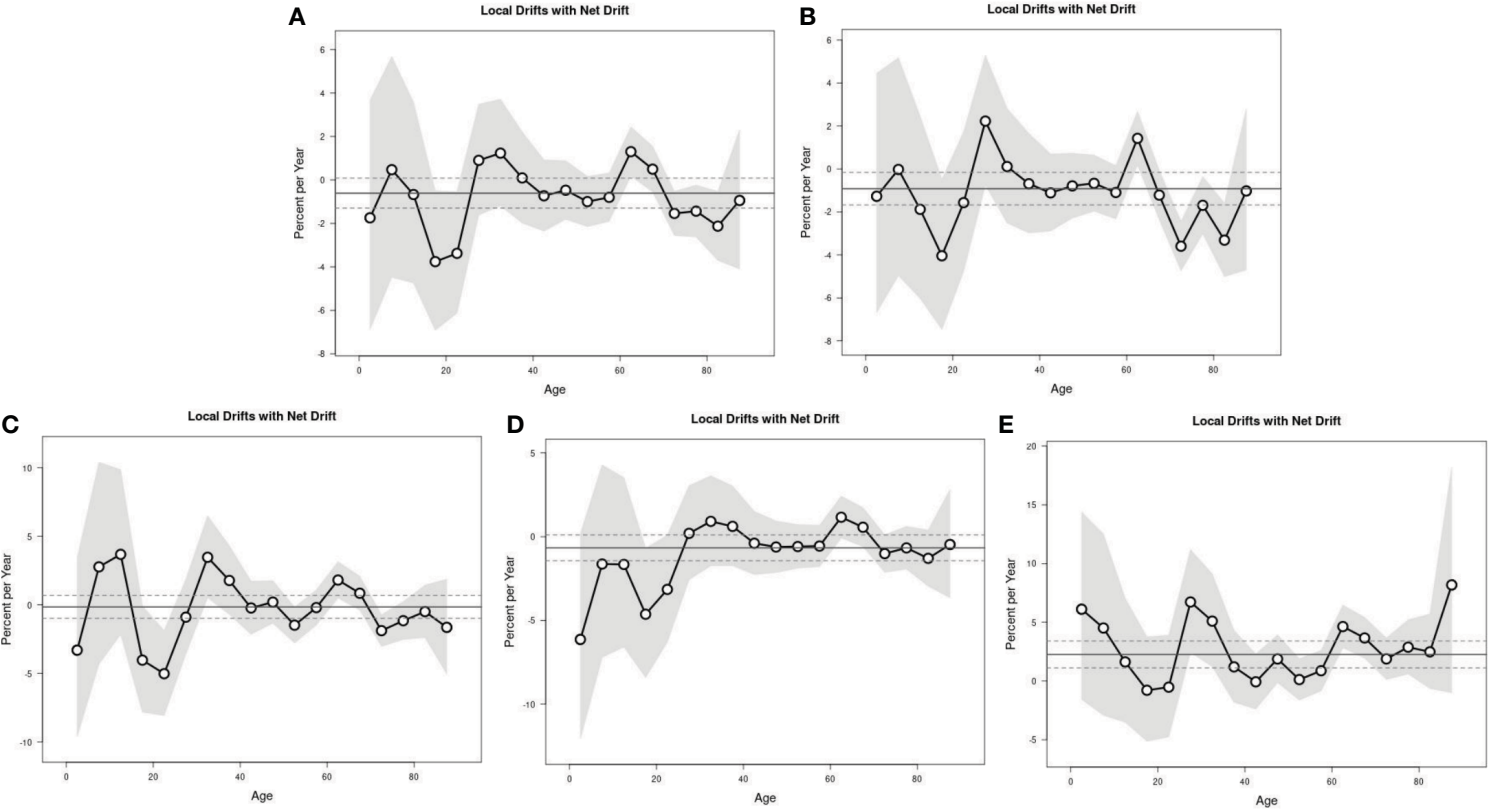
The local drifts with net drift of lymphoma incidence. Nation **(A)**, men **(B)**, women **(C)**, urban **(D)**, and rural areas **(E)**.

The Wald chi-square test results of Local Drift showed a decreasing trend in the risk of lymphoma incidence in the national level, in women, and in urban areas, but the difference was not statistically significant. The risk of lymphoma incidence in men showed a decreasing trend, while the risk of lymphoma incidence in rural areas showed an increasing trend, with a statistically significant difference. The results of Local Drift showed a trend of cohort effect on the risk of incidence in the whole nation, in different genders, and in different regions, with a statistically significant difference (**Table 3**; **Figure 4**).

**Table 3:** The net drift and test results of the age–period cohort analysis of lymphoma incidence.

		Net drift (%/year)	*χ* ^2^	df	*p*
Nation					
	Net Drift = 0	−0.61	2.97	1	0.08
	All Local Drifts = Net Drift		33.21	18	0.02
Men					
	Net Drift = 0	−0.92	5.62	1	0.02
	All Local Drifts = Net Drift		56.84	18	<0.001
Women					
	Net Drift = 0	−0.15	0.13	1	0.72
	All Local Drifts = Net Drift		47.57	18	<0.001
Urban					
	Net Drift = 0	−0.66	2.86	1	0.09
	All Local Drifts = Net Drift		21.20	18	0.27
Rural					
	Net Drift = 0	2.25	15.21	1	<0.001
	All Local Drifts = Net Drift		43.01	18	<0.001

### Prediction of lymphoma incidence in China

From the fitting results of lymphoma incidence, the fitting accuracy of the BAPC model was 82.05% for the entire nation, 81.82% for men, 82.18% for women, 82.54% for urban areas, and 81.42% for rural areas (**Table 4**). It can be seen that in predicting the incidence of leukemia, the fitting accuracy of the BAPC model was above 81%, and the predictive performance of the model was considered good, and it captured the main trends of the data with a certain degree of accuracy.

**Table 4:** Results of lymphoma model prediction efficacy in China.

	Mean absolute deviation	Mean absolute percentage error	Goodness of fit
Nation	0.70	17.95%	82.05%
Men	0.84	18.18%	81.82%
Women	0.58	17.82%	82.18%
Urban	0.78	17.46%	82.54%
Rural	0.50	18.58%	81.42%

**Figure 5:**
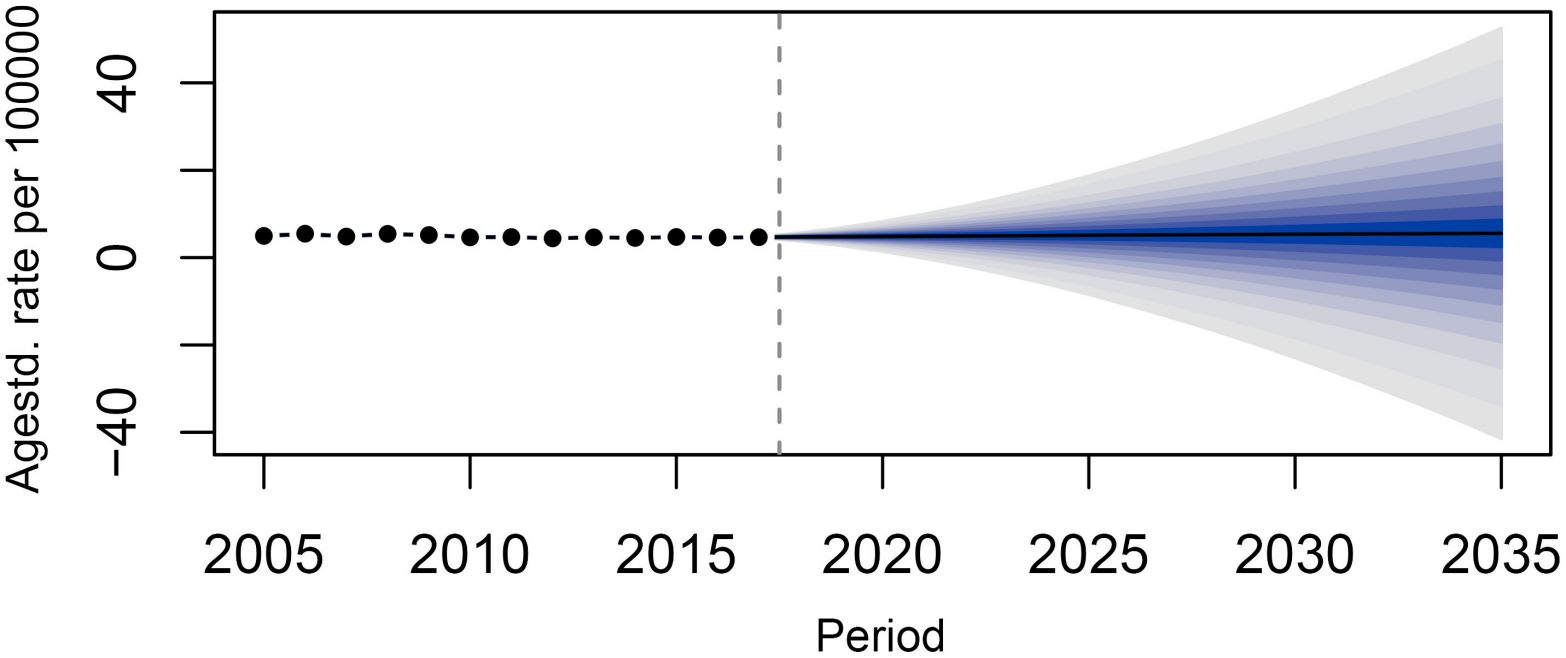
Prediction of the incidence rate of lymphoma in China from 2018 to 2035.

**Figure 6:**
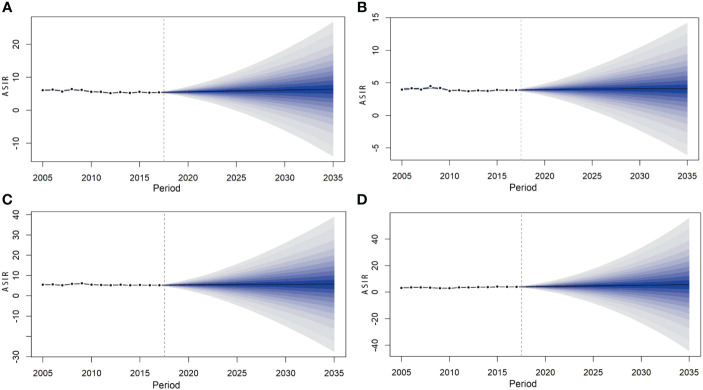
Prediction of lymphoma incidence among men **(A)**, women **(B)**, urban **(C)**, and rural areas **(D)** in China from 2018 to 2035.

The BAPC model predicted that the ASIR of lymphoma in China will continue to rise from 2018 to 2035. The incidence rate of lymphoma in 2018, 2020, 2025, 2030, and 2035 will be 4.73/100,000 (95% CI: 4.69, 4.76), 4.84/100,000 (95% CI: 4.80, 4.88), 5.11/100,000 (95% CI: 5.07, 5.15), 5.35/100,000 (95% CI: 5.31, 5.38), and 5.57/100,000 (95% CI: 4.69,4.76), respectively. The changing trend of lymphoma incidence in men and urban areas was similar to that in the whole country, while that in women and rural areas was relatively flat. It is predicted that the lymphoma incidence in men will still be higher than that in women in the future. The incidence in men and women will increase from 5.48/100,000 (95% CI: 5.42, 5.53) and 3.94/100,000 (95% CI: 3.90, 3.99) in 2018 to 6.38/100,000 (95% CI: 6.32, 6.44) and 4.09/100,000 (95% CI: 4.04, 4.14) in 2035, respectively. The incidence in urban and rural areas will increase from 5.32/100,000 (95% CI: 5.27, 5.37) and 4.12/100,000 (95% CI: 4.07, 4.17) in 2018 to 5.72/100,000 (95% CI: 5.67, 5.78) and 5.77/100,000 (95% CI: 5.72, 5.83) in 2035, respectively (**Table 5**; **Figures 5**, **6**).

**Table 5:** BAPC model predicts the incidence of lymphoma in China from 2018 to 2035 (/100,000).

Year	National		Men		Women		Urban		Rural	
	ASIR	95% CI	ASIR	95% CI	ASIR	95% CI	ASIR	95% CI	ASIR	95% CI
2018	4.73	4.69, 4.76	5.48	5.42, 5.53	3.94	3.90, 3.99	5.32	5.27, 5.37	4.12	4.07, 4.17
2019	4.78	4.75, 4.82	5.53	5.48, 5.58	3.96	3.92, 4.01	5.36	5.31, 5.41	4.20	4.15, 4.25
2020	4.84	4.80, 4.88	5.58	5.53, 5.64	3.98	3.94, 4.03	5.40	5.35, 5.45	4.29	4.24, 4.34
2021	4.89	4.86, 4.93	5.64	5.58, 5.69	4.00	3.95, 4.05	5.43	5.38, 5.49	4.38	4.33, 4.43
2022	4.95	4.91, 4.98	5.69	5.64, 5.75	4.02	3.97, 4.06	5.47	5.41, 5.52	4.47	4.42, 4.52
2023	5.00	4.96, 5.04	5.75	5.69, 5.80	4.03	3.99, 4.08	5.50	5.45, 5.55	4.55	4.50, 4.61
2024	5.05	5.02, 5.09	5.80	5.75, 5.86	4.05	4.00, 4.10	5.53	5.48, 5.58	4.64	4.59, 4.69
2025	5.11	5.07, 5.15	5.86	5.80, 5.91	4.06	4.01, 4.11	5.56	5.51, 5.61	4.74	4.68, 4.79
2026	5.16	5.12, 5.20	5.91	5.86, 5.97	4.07	4.02, 4.12	5.59	5.53, 5.64	4.83	4.78, 4.88
2027	5.20	5.17, 5.24	5.96	5.91, 6.02	4.08	4.03, 4.12	5.61	5.56, 5.66	4.92	4.87, 4.98
2028	5.25	5.21, 5.29	6.02	5.96, 6.07	4.08	4.04, 4.13	5.63	5.58, 5.68	5.02	4.96, 5.07
2029	5.30	5.26, 5.34	6.07	6.01, 6.13	4.09	4.04, 4.14	5.65	5.60, 5.70	5.11	5.06, 5.17
2030	5.35	5.31, 5.38	6.12	6.07, 6.18	4.09	4.05, 4.14	5.67	5.61, 5.72	5.22	5.16, 5.27
2031	5.39	5.36, 5.43	6.17	6.12, 6.23	4.10	4.05, 4.14	5.68	5.63, 5.74	5.32	5.27, 5.38
2032	5.44	5.40, 5.48	6.23	6.17, 6.28	4.10	4.05, 4.14	5.70	5.64, 5.75	5.43	5.37, 5.49
2033	5.48	5.44, 5.52	6.28	6.22, 6.34	4.10	4.05, 4.14	5.71	5.65, 5.76	5.54	5.48, 5.59
2034	5.52	5.48, 5.56	6.33	6.27, 6.39	4.09	4.05, 4.14	5.72	5.66, 5.77	5.65	5.59, 5.71
2035	5.57	5.53, 5.61	6.38	6.32, 6.44	4.09	4.04, 4.14	5.72	5.67, 5.78	5.77	5.72, 5.83

BAPC, Bayesian age–period–cohort; ASIR, age-standardized incidence rate; 95% CI, 95% confidence interval.

## Discussion

By analyzing the epidemic characteristics, age–period–cohort effect, and prediction of lymphoma from 2005 to 2035, this study found that the ASIR of men was higher than that of women, and the ASIR of rural areas was lower than that of urban areas, but the AAPC of rural areas was much higher, and the growth rate was faster. The age effect on the incidence of lymphoma all showed that the risk increased with age. In the period effect, the incidence of lymphoma in rural areas decreased first and then increased with 2010 as the cutoff point. The overall risk of lymphoma incidence was higher in the cohort before the 1970–1974 birth cohort than in the cohort after. It is predicted that from 2018 to 2035, the incidence rate of lymphoma in China, in men and women, and in urban areas will show an upward trend.

The occurrence of lymphoma is the result of multiple factors, including genetic factors, gene mutations, immune regulation imbalance, and inflammatory response (31, 32). These mechanisms lead to abnormal proliferation and differentiation of lymphocytes, eventually forming lymphoma. Some viruses, bacterial infections, and long-term exposure to certain chemicals, radiation, or harmful substances in the environment may also increase the risk of lymphoma. As previous studies have shown, infections such as Epstein–Barr virus and human immunodeficiency virus are associated with an increased incidence of lymphoma (33, 34); *Helicobacter pylori* infection is associated with gastric mucosa-related lymphoid tissue lymphoma (35).

The incidence of lymphoma was on the rise from 2005 to 2017, consistent with the results of previous studies. The increased incidence of lymphoma may be attributed to improved diagnosis and an increase in associated risk factors (36). The main principle of the fourth edition of Hematopoietic Lymphoid Tissue Tumor Classification published by WHO in 2008 (37) is to classify lymphomas into precursor cell lymphomas and mature cell lymphomas according to the cell origin, morphology, immunophenotype, genetics, and clinical characteristics of the cancer. The 2016 update of the WHO Classification of Hematopoietic and Lymphoid Tissue Tumors builds on the 2008 version by adding molecular genetic criteria (38) to make the diagnosis of lymphoma more accurate and specific (39). Moreover, this phenomenon can be partly attributed to population growth and aging. Lymphoma is very common in middle-aged and elderly people. According to a report by China’s National Bureau of Statistics, the country’s population increased from 129,533 million in 2000 to 1,412.12 million in 2020, while the proportion of people aged 65 and above increased from 6.96% to 13.50%. The aging process is significantly accelerated (7, 40). This reminds us of the need to further investigate the relationship between lymphoma and changes in population size and age composition in the future.

The occurrence of lymphoma varies by sex. The results of a study investigating the global burden of hematological malignancies from 1990 to 2019 have revealed significant gender differences in the incidence of lymphoma (41). A study in the United States showed that the incidence of lymphoma was approximately 50% higher in men than in women in 2016 (42). In an observational study of lymphoma and myeloma mortality in China from 2004 to 2017, Zhu et al. (43) also found that the mortality rate for men was almost twice that for women. In this study, the ASIR was higher in men than in women. This trend may be attributed to differences in hormonal and genetic factors between men and women, as well as differences in infections, environmental exposures, and lifestyle factors that may increase the risk of malignant blood diseases in men (36, 44). Studies have shown that smoking is one of the major risk factors for cancer in China, second only to chronic infections, accounting for 22.6% of cancer deaths (45). According to the Chinese Center for Disease Control and Prevention’s adult tobacco survey report, the current smoking rate in China is 52.1% for men and 2.7% for women (46). The fact that smoking rates are much higher among men than women may be an important contributing factor (47). Although China has made some progress in recent years, stronger tobacco control policies are still needed. Studies showed that if China strictly implements the policies recommended by the WHO Framework Convention on Tobacco Control, more than 10 million deaths are expected to be avoided by 2050 (48). Taxes and smoke-free policies are two effective interventions. Therefore, national legislation should be developed and improved as soon as possible to ban smoking in public places, include smoking cessation drugs in health insurance, and increase tobacco taxes (a 10% increase in the price of tobacco can reduce tobacco consumption in high-income countries) (49, 50).

The results of this study showed that ASIR was higher in urban areas than in rural areas, but the increase in rural areas was 2.59 times that in urban areas. This is similar to previous findings (43, 51). One possible reason is the imbalance between urban and rural development, as well as the uneven level of diagnosis, accessibility, and equity of health services. Diagnosis levels and access to health services are relatively high in urban areas, and lymphoma detection rates are higher than in rural areas. The incidence of lymphoma was increasing at a faster rate in rural areas than in urban areas, which may be related to improved access to medical services in rural areas (such as increased patient visits, improved diagnosis and detection rates), expansion and refinement of cancer registries, and other factors. For example, access to medical services in rural areas continues to improve, and in 2009, China launched a major healthcare reform to provide equal access to basic medical services for all citizens. Since then, government spending on healthcare has increased fourfold (52). From 2008 to 2014, health resources and access in rural areas increased by approximately 50% (53). In 2008, the central government provided financial support to launch a national cancer registration project, and the coverage and number of people are constantly expanding. By 2014, there were 449 cancer registries nationwide, covering approximately 25% of the country’s population. The implementation of the Measures for the Administration of Cancer Registration in 2015 has improved the cancer registration statistical system (14, 54). These factors may lead to increased incidence.

The incidence of lymphoma in China, different genders, and different regions showed an increasing trend. The reasons for this may be the aging of the population, environmental factors, and increased social support (e.g., increased prevalence of early diagnosis and treatment programs for cancer), which further increase the detection rate of cancer and raise the incidence rate of the cancer. For example, with the aging trend of the global population intensifying, the incidence of lymphoma is relatively high in the elderly population. By 2035, the elderly population is projected to increase, which could lead to an increase in the incidence of lymphoma. By 2035, environmental pollution is likely to increase due to continued industrialization and urbanization, including air, water, and soil pollution, which may increase the risk of lymphoma.

In this study, the BAPC method was used for prediction because of its wide acceptance in public health research and its utility for analyzing long-term trends and making future projections. The BAPC model uses INLA, and its main advantage is that it directly approximates the posterior marginal distributions without the need for convergence diagnostics. The parameter estimation is performed using the BAPC package, which not only eliminates the need for complicated coding processes and convergence checking, but also provides the flexibility to choose the parameter of standardized incidence or standardized mortality, and the *a priori* probability distribution as needed (55, 56). Studies have shown that the BAPC model has a higher accuracy in predicting cancer burden than other models and is able to obtain more reasonable and robust results, especially in short- and medium-term prediction years. Therefore, the BAPC model was selected for predictive analysis in this study (57, 58).

There are some limitations to this study. The lymphoma data were all from the 2008–2020 “Chinese Cancer Registry Annual Report”. The original data came from the National Cancer Registry instead of random sampling, so the representativeness and extrapolation results of the whole population are lacking. This study also has limitations in terms of timeliness. The latest cancer registration data are generally delayed by 3 years. Subtypes of lymphoma were not examined in this study due to insufficient available information. Without a breakdown to specific subtypes, the analysis could not reveal epidemiological details of specific subtypes of lymphoma, such as trends and differences in incidence rates for specific subtypes. However, this study provided a macroscopic view of the overall incidence and mortality of lymphomas, which helps to understand the epidemiological characteristics of lymphomas as an overall category in a specific region or population. Such macro-analysis may still be of great value for public health planning and resource allocation. The BAPC model, although widely used in predicting disease burden, has limitations, mainly including linear assumptions about age, period, and cohort effects, and failure to directly incorporate changes in risk factors and covariates. For example, the BAPC model is unable to take into account the effects of population aging, policies, genetics, and the environment, and therefore has a negative impact on the accuracy and reliability of quantitative forecasts. We are currently collecting and organizing relevant spatial data, and future studies may further explore these factors with a view to improving the accuracy of the predictions.

In summary, this study conducted trend analysis, age–period–cohort analysis, and prediction based on lymphoma data and found that the incidence of lymphoma increased from 2005 to 2017, and the risk was related to the region, gender, and age, and the incidence of lymphoma was predicted to decline slowly by 2035. The annual incidence of lymphoma showed a slow downward trend. This reminds us that we still need to pay attention to the prevention and treatment of lymphoma. The results of this study provide information on the disease burden of lymphoma, which will help formulate policies for lymphoma prevention, control, and management strategies, and have important reference significance for lymphoma prevention and treatment in China.

## Data availability statement

The original contributions presented in the study are publicly available. This data can be found here: Chinese Cancer Registry Annual Report, 2008-2020.

## Author contributions

KL: Conceptualization, Formal analysis, Methodology, Software, Writing – original draft, Writing – review & editing. JS: Conceptualization, Formal analysis, Methodology, Software, Writing – original draft, Writing – review & editing. YC: Conceptualization, Formal analysis, Methodology, Software, Writing – original draft, Writing – review & editing. LL: Formal analysis, Methodology, Software, Writing – review & editing. PL: Formal analysis, Methodology, Software, Writing – review & editing. XC: Formal analysis, Methodology, Software, Writing – review & editing. MT: Formal analysis, Methodology, Software, Writing – review & editing. YL: Formal analysis, Methodology, Software, Writing – review & editing. YY: Conceptualization, Formal analysis, Funding acquisition, Methodology, Project administration, Software, Supervision, Writing – original draft, Writing – review & editing. LZ: Conceptualization, Formal analysis, Funding acquisition, Methodology, Project administration, Resources, Software, Supervision, Writing – original draft, Writing – review & editing. XP: Conceptualization, Formal analysis, Funding acquisition, Methodology, Project administration, Resources, Software, Supervision, Writing – original draft, Writing – review & editing. WN: Project administration, Resources, Software, Supervision, Writing – original draft, Writing – review & editing, Conceptualization, Formal analysis, Funding acquisition, Methodology.
